# Multicenter Clinical Comparison Between Anti‐NXP2 and Anti‐TIF1‐γ Antibody‐Positive Myositis, and Analysis of the Clinical Significance of Anti‐NXP2 Antibody Titers in Idiopathic Inflammatory Myopathies

**DOI:** 10.1111/1346-8138.70490

**Published:** 2026-09-16

**Authors:** Satoshi Kamiya, Mariko Ogawa‐Momohara, Yoshinao Muro, Yuta Yamashita, Satoko Yuasa, Masato Kakeda, Kimiko Nakajima, Hiroyuki Morita, Mitsuyo Kinjo, Takako Hashimoto, Ken Yoshida, Takuya Takeichi, Haruka Koizumi, Norika Akashi, Eori Noda, Masashi Akiyama

**Affiliations:** ^1^ Department of Dermatology Nagoya University Graduate School of Medicine Nagoya Aichi Japan; ^2^ Department of Dermatology Fujita Health University School of Medicine Toyoake Aichi Japan; ^3^ Department of Dermatology Saiseikai Matsusaka General Hospital Matsusaka Mie Japan; ^4^ Department of Dermatology Mie University Graduate School of Medicine Tsu Mie Japan; ^5^ Department of Dermatology Kochi Medical School, Kochi University Nankoku Kochi Japan; ^6^ Department of General Internal Medicine Gifu University Graduate School of Medicine Gifu Japan; ^7^ Department of Internal Medicine Gifu Prefectural Gero Hospital Gero, Gifu Japan; ^8^ Division of Rheumatology, Department of Medicine Okinawa Chubu Hospital Okinawa Japan; ^9^ Division of Rheumatology, Department of Internal Medicine Fujita Health University School of Medicine Toyoake Aichi Japan; ^10^ Division of Rheumatology, Department of Internal Medicine Jikei University School of Medicine Tokyo Japan; ^11^ Department of Dermatology Toyota Memorial Hospital Toyota Aichi Japan; ^12^ Department of Dermatology Shin‐Yurigaoka General Hospital Kawasaki Kanagawa Japan

**Keywords:** anti‐NXP2 antibody, anti‐TIF1‐gamma antibody, cancer‐associated myositis, dermatomyositis, idiopathic inflammatory myopathy

## Abstract

We aimed to measure anti‐nuclear matrix protein 2 antibody (anti‐NXP2) titers in patients with anti‐NXP2‐positive idiopathic inflammatory myopathies, investigate whether these titers are associated with clinical manifestations, malignancy risk, and treatment response, and compare the clinical and serological features between anti‐NXP2‐positive and anti‐transcriptional intermediary factor 1‐γ antibody (anti‐TIF1‐γ)‐positive dermatomyositis in order to identify practical indicators for differentiating the two subsets. We established a newly optimized in‐house enzyme‐linked immunosorbent assay for the quantitative measurement of anti‐NXP2. After excluding two of the 34 initially identified patients with anti‐NXP2 antibody reactivity, we used the assay to analyse sera from 32 patients with anti‐NXP2‐positive idiopathic inflammatory myopathy. Clinical correlations between anti‐NXP2 titers and inflammatory indices, cardiothoracic ratio, malignancy status, and longitudinal changes after immunosuppressive therapy were examined. We developed a novel clinical scoring system to differentiate between anti‐NXP2‐positive and anti‐TIF1‐γ‐positive myositis. The anti‐TIF1‐γ‐positive patients showed a higher frequency of classic dermatomyositis skin manifestations, whereas the anti‐NXP2‐positive patients had significantly higher creatine kinase, aldolase, and lactate dehydrogenase levels, indicating more prominent muscle involvement. Anti‐NXP2 titers correlated with erythrocyte sedimentation rate and cardiothoracic ratio and were significantly higher in patients with malignancy in the anti‐NXP2‐positive idiopathic inflammatory myopathy patients. Longitudinal analyses demonstrated declines in antibody titers after immunosuppressive treatment. Our scoring system, incorporating age at onset, Gottron's sign, creatine kinase, lactate dehydrogenase, and malignancy status, highly accurately distinguished between anti‐NXP2‐positive and anti‐TIF1‐γ‐positive myositis. Anti‐NXP2 titers reflect systemic inflammation, cardiac involvement, and malignancy risk in anti‐NXP2‐positive idiopathic inflammatory myopathy. The observed decline in anti‐NXP2 titers after therapy suggests the potential utility of these titers as biomarkers for longitudinal disease assessment and stratifying malignancy risk.

## 1 Introduction

Idiopathic inflammatory myopathies (IIMs) are a heterogeneous group of autoimmune diseases primarily characterized by inflammation of the skin and skeletal muscles. Representative subtypes include dermatomyositis (DM), polymyositis (PM), immune‐mediated necrotizing myopathy (IMNM), and inclusion body myositis (IBM). It is well established that the presence and specificity of myositis‐specific autoantibodies (MSAs) define distinct clinical phenotypes in IIMs [1].

Anti‐nuclear matrix protein 2 (NXP2) antibodies (anti‐NXP2, also known as anti‐MJ antibodies) were first identified in juvenile dermatomyositis (JDM) [2]. Subsequent studies have demonstrated their presence across the spectrum of IIMs, including adult DM, and they are now recognized as MSAs that are predominantly associated with DM. Patients with anti‐NXP2 have been reported to exhibit severe clinical manifestations, such as marked muscle weakness and dysphagia, as well as characteristic cutaneous features including generalized edema and calcinosis [3, 4]. In addition, interstitial lung disease (ILD) has been reported to occur in a subset of IIM patients who are positive for anti‐NXP2 [5].

In contrast, anti‐transcriptional intermediary factor 1‐γ (TIF1‐γ) antibodies (anti‐TIF1‐γ, also referred to as anti‐p155/140 antibodies) are characterized by typical DM‐like skin eruptions and are strongly associated with malignancy in adult DM [6]. Both anti‐NXP2 and anti‐TIF1‐γ target nuclear antigens [7] and share certain clinical features [8], including the presence of DM‐specific skin manifestations such as heliotrope rash and Gottron's papules [9, 10], a high prevalence in juvenile DM [10], and a relatively low frequency of ILD compared with anti‐MDA5 or anti‐ARS antibody‐positive myositis. Conversely, both antibodies are associated with a higher prevalence of malignancy. While anti‐TIF1‐γ antibodies are well established as a hallmark of cancer‐associated DM (CA‐DM), anti‐NXP2 has also been linked to malignancy in adult DM and are increasingly recognized as an important MSA when considering CA‐DM [10, 11].

Most previous studies have focused on the presence or absence of MSAs or on comparisons of clinical features among different MSA subtypes. However, no study has quantitatively evaluated anti‐NXP2 and systematically examined the association between this antibody and clinical manifestations. In this study, we measured anti‐NXP2 titers by using our newly optimized ELISA system in patients with anti‐NXP2‐positive IIM to clarify the clinical significance of this antibody. Furthermore, through comparative analyses with anti‐TIF1‐γ‐positive DM patients, we aimed to delineate differences in patient characteristics, cutaneous manifestations, and serological findings, and to identify clinically useful indicators for distinguishing between these two subsets.

## 2 Materials and Methods

### 2.1 Study Population and Clinical Samples

This multicenter retrospective study includes serum samples from patients evaluated at multiple participating institutions and stored in the serum bank of the Department of Dermatology, Nagoya University Hospital. A total of 73 patients with IIM were included, comprising 34 patients positive for anti‐NXP2 identified using our established detection methods [12] and 39 patients positive for anti‐TIF1‐γ antibodies identified using a commercial ELISA kit (MBL, Japan). Among the 34 patients who were initially identified as anti‐NXP2‐positive, two were excluded from the final comparative analysis because their anti‐NXP2 reactivity was equivocal and was not confirmed as definite positivity by IPP. Therefore, patients for the final comparative analysis consisted of 32 anti‐NXP2‐positive patients and 39 anti‐TIF1‐γ‐positive patients. Among the 34 initially identified anti‐NXP2‐positive patients, the disease distribution comprised 31 cases of DM (including three of juvenile DM and two of clinically amyopathic DM [CADM]), two cases of polymyositis (PM), and one case of IMNM. Four patients were classified as having cancer‐associated myositis (CAM). Serum samples were collected at the time of diagnosis. These samples from 35 healthy Japanese individuals were included as normal controls. The samples from the anti‐NXP2‐positive patients were collected from multiple departments, including Dermatology (*n* = 22), Neurology (*n* = 1), Rheumatology (*n* = 1), Rheumatology and Collagen Diseases (*n* = 6), General Internal Medicine (*n* = 3), and Pediatrics (*n* = 1).

Patients with DM and PM fulfilled the Bohan and Peter criteria [13, 14]. CADM was diagnosed according to Sontheimer's criteria [15], and patients who developed ILD within 6 months of disease onset were included in the CADM group. JDM was defined as disease onset before the age of 16 years based on the Bohan and Peter criteria [16]. CAM was defined as the diagnosis of internal malignancy within 3 years before or after the diagnosis of DM or PM, in accordance with previous studies [17].

Clinical data were retrospectively collected from medical records using standardized case report forms. Collected variables included sex, age at onset, diagnosis, date of serum sampling, cutaneous manifestations (heliotrope rash, Gottron's papules/sign, V‐neck sign, shawl sign, flagellate erythema, periungual erythema, skin ulcers, calcinosis, mechanic's hands, poikiloderma, and alopecia), extracutaneous features (fever, pruritus, dysphagia, arthralgia, myositis, ILD, associated autoantibodies, malignancy), cardiovascular events, treatment interventions, and laboratory findings (serum creatine kinase [CK], lactate dehydrogenase [LDH], aldolase, ferritin, erythrocyte sedimentation rate [ESR], among others).

ILD was diagnosed based on fibrotic changes detected on chest radiography or computed tomography. Malignancy screening performed at or around the time of myositis diagnosis included whole‐body CT, upper and lower gastrointestinal endoscopy, gynecological examination, and breast cancer screening. Malignancy status was assessed by reviewing the results of these screening examinations as well as subsequent malignancy development during longitudinal clinical follow‐up. The timing of malignancy detection and cancer types was collected from medical records when available. All participants provided written informed consent for study participation, including serum use. In selected cases, longitudinally stored serum samples were used to evaluate changes in antibody titers over time. This study was approved by the Ethics Committee of Nagoya University Graduate School of Medicine and conducted in accordance with the Declaration of Helsinki.

### 2.2 Development of Newly Optimized Enzyme‐Linked Immunosorbent Assay (ELISA) for Anti‐NXP2

An ELISA for the detection of anti‐NXP2 was previously established in our department [12]. In the present cohort, several sera exceeded the upper detection limit of the conventional assay. Therefore, we newly optimized a quantitative ELISA using high‐titer reference sera to enable accurate measurement across a broader dynamic range. The quantification of anti‐NXP2 was performed using a solid‐phase direct ELISA based on a previously established in‐house method, which measures the specific binding of serum antibodies to recombinant NXP2 protein. The cutoff value was defined as the mean plus three standard deviations of 35 healthy control sera, yielding a cutoff of 1.19 U.

To minimize nonspecific binding, the assay was modified by incorporating a biotinylated transcription and translation (TnT) product unrelated to the target antigen as a control antigen (DACA1), allowing discrimination between background signals and anti‐NXP2‐specific reactivity.

### 2.3 Immunoprecipitation (IPP) Assay

Immunoprecipitation was performed using TnT products with minor modifications, as previously described [18, 19, 20, 21]. Details of the assay procedures and validation of the measurement system are provided in the Supplementary Methods and Figure S1. The IPP assay was used to confirm anti‐NXP2 reactivity, particularly in sera with low‐positive or equivocal ELISA results. Representative IPP findings, including low‐positive, equivocal, ELISA‐negative, and healthy control sera, are shown in Figure S1.

### 2.4 Statistical Analysis

Antibody positivity rates were compared using Fisher's exact test. Associations between clinical features and antibody status were analysed using Fisher's exact test for categorical variables and the Mann–Whitney U test for continuous variables, as appropriate. A clinical scoring system was developed to discriminate between patient subsets with specific antibodies and evaluated by receiver operating characteristic (ROC) curve analysis. Cutoff values for continuous variables included in the clinical scoring system were determined by ROC curve analysis using anti‐NXP2 antibody positivity as the outcome. For each variable, the optimal cutoff value was selected based on the Youden index, defined as sensitivity + specificity −1. Correlations between anti‐NXP2 titers and clinical variables were assessed using Spearman's rank correlation coefficient. Statistical analyses were performed using GraphPad Prism version 9.5.1 and SPSS Statistics version 31.0, with *p* < 0.05 considered statistically significant.

## 3 Results

### 3.1 Comparison of Clinical Characteristics Between Anti‐NXP2‐Positive IIM and Anti‐TIF1‐γ‐Positive IIM

We compared the major clinical and laboratory features of 32 anti‐NXP2‐positive patients and 39 anti‐TIF1‐γ‐positive patients (Table 1). The median age at onset was significantly younger in the anti‐NXP2‐positive group (48 years) than in the anti‐TIF1‐γ‐positive group (63 years; *p* = 0.0060).

**TABLE 1:** Comparative clinical characteristics of patients with anti‐NXP2 and anti‐TIF1‐γ‐positive idiopathic inflammatory myopathies.

Characteristics	anti‐NXP2^+^ patients (*n* = 32)	anti‐TIF1‐γ^+^ patients (*n* = 39)	*p*
Clinical group			
DM, *n* (%)	24 (75.0)	27 (69.2)	0.79
CADM, *n* (%)	2 (6.25)	10 (25.6)	0.053
JDM, *n* (%)	3 (9.38)	2 (5.13)	0.65
PM, *n* (%)	2 (6.25)	0 (0)	0.20
IMNM, *n* (%)	1 (3.13)	0 (0)	0.45
Demographic features			
Age at onset (years), median (IQR)	48.0 (32.5–56.5)	63.0 (51.0–69.0)	0.0060**
Female gender, *n* (%)	24/32 (75.0)	26/39 (66.7)	0.74
Cutaneous manifestations			
Gottron's sign/papules, *n* (%)	16/29 (55.2)	30/34 (88.2)	0.0044**
Heliotrope rash, *n* (%)	10/29 (34.5)	26/34 (76.5)	0.0010**
V‐neck sign, *n* (%)	10/29 (34.5)	16/34 (47.1)	0.44
Shawl sign, *n* (%)	14/29 (48.3)	12/34 (35.3)	0.32
Mechanic's hands, *n* (%)	3/29 (10.3)	5/34 (14.7)	0.72
Periungual erythema, *n* (%)	15/29 (51.7)	25/34 (73.5)	0.11
Flagellate erythema, *n* (%)	3/29 (10.3)	3/34 (8.8)	1
Poikiloderma, *n* (%)	4/29 (13.8)	1/34 (2.9)	1
Alopecia, *n* (%)	1/29 (3.4)	2/34 (5.9)	1
Skin ulceration, *n* (%)	4/29 (13.8)	5/34 (14.7)	1
Calcinosis, *n* (%)	4/30 (13.3)	4/34 (11.8)	1
Extracutaneous manifestations			
Pruritus, *n* (%)	9/29 (31.0)	12/34 (35.3)	0.79
Fever, *n* (%)	6/29 (20.7)	4/34 (11.8)	0.49
Arthritis, *n* (%)	6/20 (30.0)	6/33 (18.2)	0.36
Myositis, *n* (%)	27/29 (93.1)	25/33 (75.8)	0.092
Dysphagia, *n* (%)	9/29 (31.0)	11/34 (32.4)	1
Interstitial lung disease, *n* (%)	4/29 (13.8)	3/31 (9.7)	0.70
Cancer, *n* (%)	4/29 (13.8)	21/38 (55.2)	0.00076****
Cardiothoracic ratio (%), median	48.2, (*n* = 23)	46.5, (*n* = 29)	0.10
Abnormal echocardiographic findings, *n* (%)	6/17 (35.3)	12/32 (37.5)	1
Electrocardiographic abnormalities, *n* (%)	8/29 (27.6)	11/24 (45.8)	0.25
Coexisting autoantibodies, *n* (%)	8/29 (27.6)	7/34 (20.6)	0.56
Treatment initiated, *n* (%)	7/29 (24.1)	5/32 (14.3)	0.52
Laboratory testing results, median (IQR)			
WBC	6150.0 (4250.0–7650.0), (*n* = 31)	6000.0 (4600.0–7550.0), (*n* = 38)	0.79
Eosinophils, %	1.9 (0.7–3.5), (*n* = 31)	1.9 (0.9–4.0), (*n* = 32)	0.50
Eosinophil count	108.4 (45.8–198.5), (*n* = 31)	95.6 (54.0–228.0), (*n* = 32)	0.51
Lymphocytes, %	19.8 (10.8–27.7), (*n* = 29)	18.0 (14.8–27.2), (*n* = 33)	0.84
Lymphocyte count	1086.9 (683.4–1459.6), (*n* = 29)	1008.1 (712.8–1592.8), (*n* = 33)	0.81
Hb	13.2 (12.1–15.1), (*n* = 29)	12.5 (11.8–13.6), (*n* = 37)	0.18
Plt	24.3 (18.4–28.7), (*n* = 31)	20.7 (18.1–26.7), (*n* = 37)	0.3045
LDH	388.0 (278.0–516.0), (*n* = 30)	303.0 (220.8–395.8), (*n* = 35)	0.0092**
CK	723.0 (180.8–1823.2), (*n* = 31)	250.0 (74.0–625.0), (*n* = 33)	0.023*
Aldolase	13.0 (8.5–16.8), (*n* = 29)	8.6 (5.3–10.8), (*n* = 33)	0.0026***
Myoglobin	134.1 (41.9–467.2), (*n* = 24)	98.2 (27.9–227.9), (*n* = 23)	0.20
CK–MB	21.8 (11.8–105.6), (*n* = 14)	25.0 (20.5–29.0), (*n* = 6)	0.84
ESR	21.5 (12.0–29.0), (*n* = 25)	12.0 (9.0–19.0), (*n* = 14)	0.36
Potassium	4.1 (3.9–4.3), (*n* = 29)	4.1 (4.0–4.3), (*n* = 35)	0.80
KL‐6_max_	216.0 (199.0–313.0), (*n* = 24)	226.5 (208.0–347.0), (*n* = 24)	0.079
SP‐D_max_	43.2 (29.8–63.4), (*n* = 12)	51.1 (36.0–67.0), (*n* = 14)	0.37
Ferritin_max_	159.4 (18.5–902.0), (*n* = 21)	145.5 (91.8–317.0), (*n* = 14)	0.23
Troponin T_max_	0.044 (0.010–0.070), (*n* = 24)	0.018 (0.0048–0.050), (*n* = 20)	0.27

*Note:* Demographic, clinical, and laboratory characteristics of patients with anti‐NXP2‐positive and anti‐TIF1‐γ‐positive IIMs are summarized. Age at onset, sex, cutaneous and extracutaneous manifestations, malignancy status, and serum biomarkers are compared between groups. Note that percentages may not reflect the number divided by the total number of subjects when data are missing. Values are presented as number (%) or median (interquartile range), unless otherwise indicated. *p* values for categorical variables were calculated using Fisher's exact test. For each clinical subgroup, the frequency of that subgroup was compared between the two antibody groups using a 2 × 2 contingency table. *p* values for continuous variables were calculated using the Mann–Whitney U test.

Abbreviations: CK, creatine kinase; CK–MB, creatine kinase–MB; ESR, erythrocyte sedimentation rate; Hb, hemoglobin; IQR, interquartile range; KL‐6, Krebs von den Lungen‐6; LDH, lactate dehydrogenase; Plt, platelets; SP–D, surfactant protein‐D; TIF1‐γ, transcription intermediary factor 1‐γ; WBC, white blood cell count.

Significant differences: *p ≤ 0.05, **p ≤ 0.01, ***p ≤ 0.005, ****p ≤ 0.001.

Regarding cutaneous manifestations, classic DM skin findings such as Gottron's sign/papules and heliotrope rash were more frequent in the anti‐TIF1‐γ‐positive group (Gottron's sign/papules: 88.2%; heliotrope rash: 76.5%) than in the anti‐NXP2‐positive group (55.2% and 34.5%, respectively). No clear differences were observed between the two groups in other skin findings, including V‐neck sign, shawl sign, periungual erythema, flagellate erythema, poikiloderma, alopecia, skin ulcers, or calcinosis.

The frequencies of myositis, arthralgia, or dysphagia did not differ significantly between groups. In laboratory findings at the initial visit, serum CK, LDH, and aldolase levels were significantly higher in the anti‐NXP2‐positive group (*p* = 0.023, 0.0092, and 0.0026, respectively).

For organ involvement, we compared the prevalence of ILD, serum KL‐6 and SP‐D, cardiac troponin T, and abnormalities on electrocardiography and echocardiography, but no significant differences were identified in any of these items between the two groups.

Malignancy was significantly more common in the anti‐TIF1‐γ‐positive group (*p* < 0.001). In the anti‐NXP2‐positive group, malignancy was observed in only four patients (13.8%). Of these, two patients were diagnosed within approximately one month after myositis diagnosis, whereas the remaining two were diagnosed approximately one year after myositis diagnosis during longitudinal follow‐up. The cancer types were ovarian cancer, endometrial cancer, bladder carcinoma in situ, and lung cancer.

In the anti‐NXP2 antibody‐positive cohort, malignancy was identified in four patients during follow‐up after myositis diagnosis, with a median interval of 9.45 months from myositis diagnosis to malignancy diagnosis (IQR, 0.79–18.23 months). In contrast, malignancy in the anti‐TIF1‐γ antibody‐positive cohort was more frequent and tended to cluster around the time of myositis diagnosis; among patients with available timing information, the median interval was 0.12 months (IQR, −1.33–0.78 months). Negative values indicate cancer diagnosis before myositis diagnosis. The timings of malignancy diagnosis and cancer types are summarized in Table S1.

### 3.2 Development of a Novel Clinical Scoring System for Anti‐NXP2‐Positive IIM

Of the 32 anti‐NXP2‐positive cases and the 39 anti‐TIF1‐γ‐positive cases, 28 and 32 cases, respectively, had complete data for all five variables (based on the results of Table 1) and were included in the development of the clinical scoring system. The score was designed to reflect features characteristic of anti‐NXP2‐positive disease, and it consisted of five items (total score 0–5): age at onset (< 50 years: 1 point), absence of Gottron's sign/papules (1 point), elevated serum LDH (≥ 423 U/L: 1 point), elevated serum CK (≥ 367 U/L: 1 point), and absence of malignancy (1 point) (Figure 1A).

**FIGURE 1:**
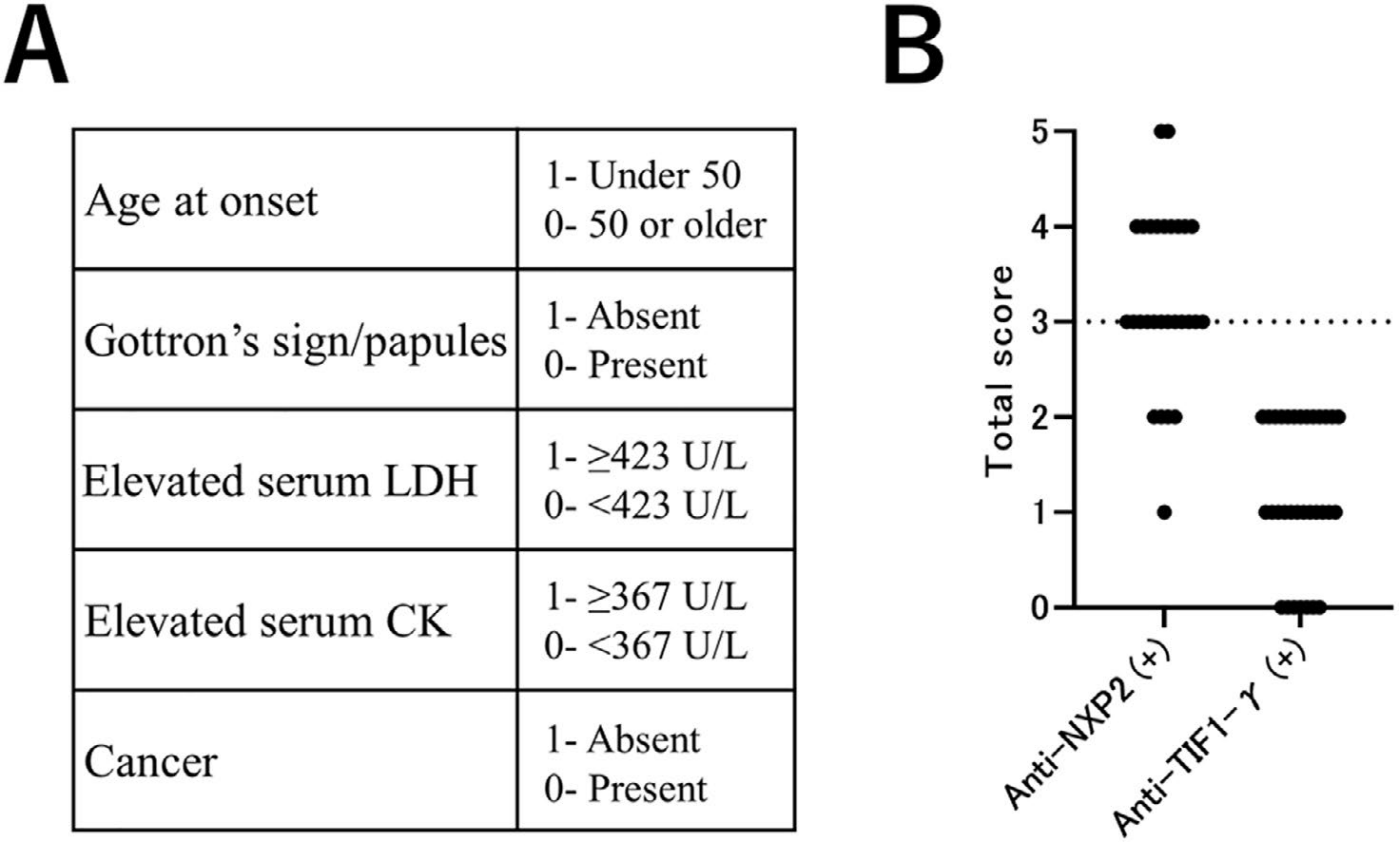
Clinical score for distinguishing anti‐NXP2‐positive myositis from anti‐TIF1‐γ‐positive dermatomyositis A. A novel clinical scoring system was constructed based on five variables: Age at onset < 50 years (1 point), absence of Gottron's sign/papules (1 point), elevated serum lactate dehydrogenase (LDH) (1 point), elevated serum creatine kinase (CK) (1 point), and absence of malignancy (1 point), yielding a total score ranging from 0 to 5. B. The clinical scoring system for identifying anti‐NXP2‐positive cases. With a cutoff value of ≥ 3 points, the score demonstrated a sensitivity of 81.5% and a specificity of 100%, with no false‐positive cases and five false‐negative cases. CK, creatine kinase; LDH, lactate dehydrogenase; NXP2, nuclear matrix protein 2; TIF1‐γ, transcriptional intermediary factor 1‐gamma.

ROC curve analysis showed that, at a cutoff score of ≥ 3, the score identified anti‐NXP2‐positive cases with a sensitivity of 82.1% and a specificity of 100% (AUC 0.950).

Using this cutoff, no false‐positive cases were observed, but five false‐negative cases were noted (Figure 1B).

### 3.3 Newly Optimized ELISA and Immunoprecipitation Assay for Anti‐NXP2

Using the newly optimized ELISA, we measured sera from 46 patients with connective tissue diseases (34 anti‐NXP2‐positive IIM, six DM, two SLE, and four SSc) as well as sera from 35 healthy controls. With the cutoff value defined as the mean + 3 standard deviations (SD) (1.19 U) (Figure 2A), 30 of the 34 anti‐NXP2‐positive IIM cases were positive by ELISA, whereas four cases were below the cutoff.

**FIGURE 2:**
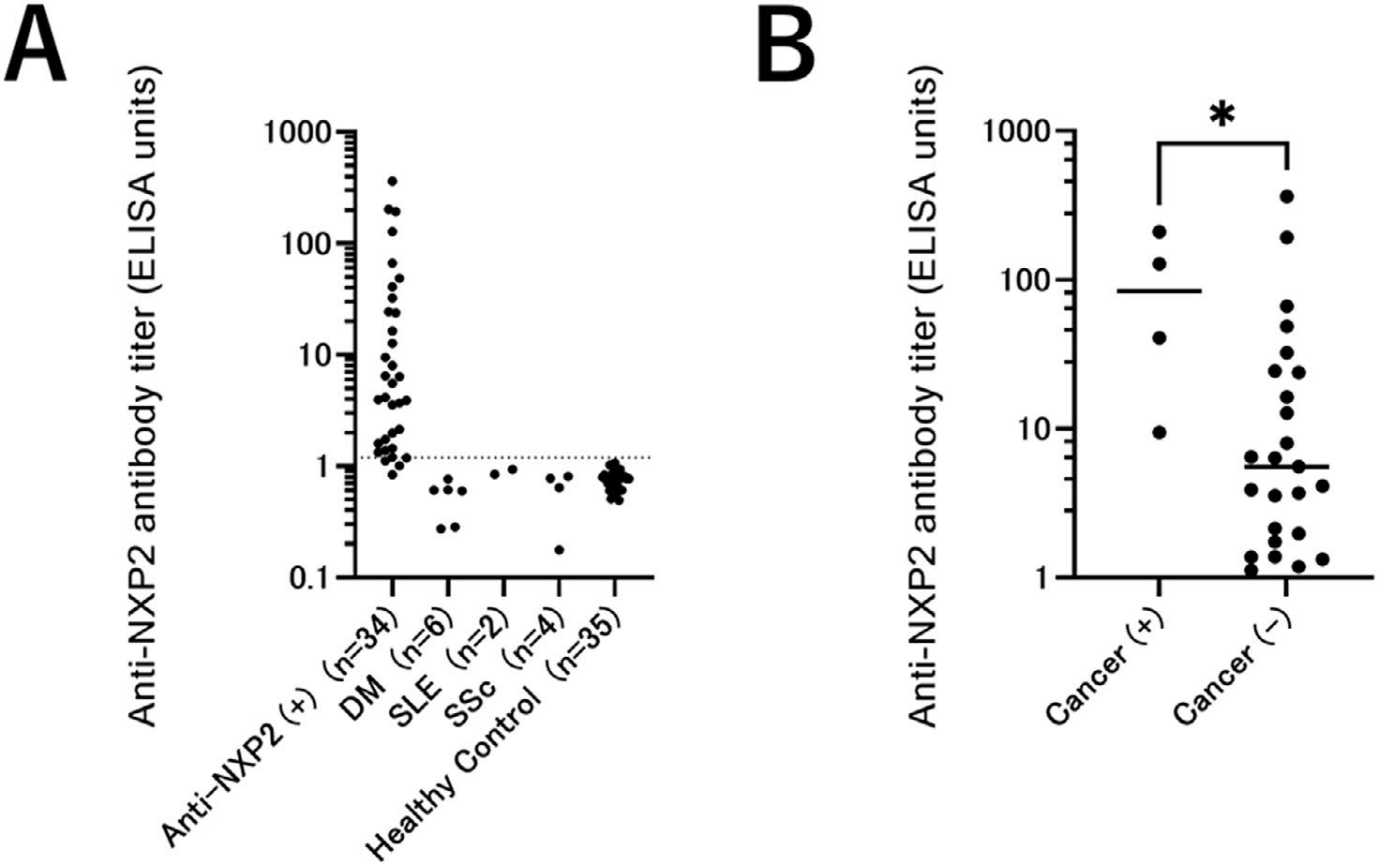
ELISA‐based quantification of anti‐NXP2 titers and association with malignancy A. Anti‐NXP2 titers were measured using our newly optimized ELISA with biotinylated recombinant NXP2 in a total of 81 serum samples: 46 from patients with connective tissue diseases and 35 from healthy controls. The cutoff value was defined as the mean + 3 SD of the healthy control group (1.19 U). B. Anti‐NXP2 titers were nominally significantly higher in patients with malignancy than in those without malignancy (median 84.15 vs. 5.55, *p* = 0.030, Mann–Whitney U test). DM, dermatomyositis; ELISA, enzyme‐linked immunosorbent assay; SLE, systemic lupus erythematosus; SSc, systemic sclerosis.

To verify whether the ELISA‐negative or borderline results near the cutoff reflected true anti‐NXP2 reactivity, we performed immunoprecipitation (IPP) for sera around the cutoff, including for these four cases.

As described above, after ELISA measurement, IPP was performed in five anti‐NXP2 ELISA‐positive cases, four equivocal cases, and three healthy control sera. Two of the four cases judged negative by ELISA exhibited a clear band at approximately 140 kDa on IPP. In contrast, none of the three healthy control sera showed reactivity (Figure S1). After ELISA screening and subsequent IPP confirmation, two initially anti‐NXP2‐positive cases were reclassified as negative; therefore, 32 anti‐NXP2‐positive patients were included in the comparative analyses.

### 3.4 Correlation Analyses Between Anti‐NXP2 ELISA Titers and Clinical/Laboratory Findings in Anti‐NXP2‐Positive IIM

Associations between anti‐NXP2 ELISA titers and major clinical or laboratory variables were assessed using Spearman correlation analysis (Table 2). Among the laboratory parameters, the maximum ESR (ESR_max_) showed a nominally significant moderate positive correlation with antibody titer (*r* = 0.51, *p* = 0.0090). The cardiothoracic ratio also showed a nominally significant positive correlation with titer (*r* = 0.49, *p* = 0.017) (Figure S2). In contrast, CK level tended to correlate negatively with titer (*r* = −0.35, *p* = 0.052). No other nominally significant correlations were observed between titers and other laboratory measures (white blood cell count, platelet count, LDH_max_, CK_max_, CK‐MB_max_, myoglobin, hemoglobin, potassium, etc.) or other clinical features.

**TABLE 2:** Spearman correlation analysis of clinical and laboratory features in anti‐NXP2‐positive IIM according to antibody titer.

Variable	Spearman's rho (*r*)	*p*
WBC, (*n* = 31)	0.17	0.37
Eosinophils, (*n* = 31)	−0.27	0.15
Eosinophil count, (*n* = 31)	−0.29	0.12
Lymphocytes, (*n* = 29)	−0.33	0.085
Lymphocyte count, (*n* = 29)	−0.24	0.21
Hb, (*n* = 29)	−0.36	0.056
Plt, (*n* = 31)	0.032	0.86
Myoglobin, (*n* = 24)	−0.07	0.75
LDH_max_, (*n* = 31)	0.10	0.59
CK_max_, (*n* = 31)	−0.26	0.15
Aldolase_max_, (*n* = 29)	−0.09	0.64
CK–MB_max_, (*n* = 13)	−0.18	0.57
ESR_max_, (*n* = 25)	0.51	0.0090**
Potassium_max_, (*n* = 28)	0.35	0.067
KL‐6_max_, (*n* = 24)	0.25	0.24
SP‐D_max_, (*n* = 12)	0.084	0.80
Ferritin_max_, (*n* = 21)	−0.039	0.87
Troponin T_max_, (*n* = 24)	0.21	0.32
Cardiothoracic ratio, (*n* = 23)	0.49	0.017*

*Note:* Correlations between quantitative anti‐NXP2 titers and clinical parameters, including inflammatory indices and cardiothoracic ratio, are shown. Spearman's rank correlation test was applied. *r* indicates Spearman's rank correlation coefficient.

Abbreviations: CK, creatine kinase; CK‐MB, creatine kinase–MB; ESR, erythrocyte sedimentation rate; Hb, hemoglobin; KL‐6, Krebs von den Lungen‐6; LDH, lactate dehydrogenase; Plt, platelets; SP‐D, surfactant protein‐D; WBC, white blood cell count.

Nominally significant correlations:*p ≤ 0.05, **p ≤ 0.01.

### 3.5 Comparison of Anti‐NXP2 ELISA Titers According to the Presence or Absence of Diverse Clinical Features in Anti‐NXP2‐Positive IIM

Finally, we evaluated associations between anti‐NXP2 ELISA titers and individual clinical features using two‐group comparisons within the anti‐NXP2‐positive cohort (Table 3). The anti‐NXP2 titers were nominally significantly higher for patients with malignancy than for those without malignancy (median 84.15 vs. 5.55; *p* = 0.030) (Figure 2B).

**TABLE 3:** Comparison of anti‐NXP2 titers between groups of patients with and without various clinical features.

Clinical feature	Percentage of patients with relevant clinical feature (%) [positive patient n/total N]	Anti‐NXP2 titer, median		*p*
		Patient group with relevant clinical feature	Patient group without relevant clinical feature	
Cutaneous manifestations				
Gottron's sign/papules	55.2 [16/29]	15	5.6	0.62
Heliotrope rash	34.5 [10/29]	24	3.9	0.069
V‐neck sign	34.5 [10/29]	9.6	6.3	0.95
Shawl sign	48.3 [14/29]	9.5	6.5	1
Mechanic's hands	10.3 [3/29]	3.5	7.2	0.56
Periungual erythema	51.7 [15/29]	9.5	4.8	0.75
Flagellate erythema	10.3 [3/29]	8.0	6.4	0.61
Poikiloderma	13.8 [4/29]	2.5	8.0	0.25
Alopecia	3.4 [1/29]	2.1	44	0.63
Skin ulceration	13.8 [4/29]	25	6.5	1
Calcinosis	13.3 [4/30]	4.8	7.2	0.70
Extracutaneous manifestations				
Pruritus	31.0 [9/29]	8.0	6.4	1
Fever	20.7 [6/29]	15	6.3	0.38
Arthritis	30.0 [6/20]	8.0	15	0.49
Myositis	93.1 [27/29]	6.5	67	0.44
Dysphagia	31.0 [9/29]	24	4.0	0.077
Interstitial lung disease	13.8 [4/29]	5.1	8.0	0.48
Cancer	13.8 [4/29]	84	5.6	0.030*
Coexisting autoantibodies	27.6 [8/29]	5.1	6.5	0.68
Electrocardiographic abnormalities	27.6 [8/29]	7.2	6.5	0.90
Abnormal echocardiographic findings	35.3 [6/17]	24	5.6	0.40
Cardiothoracic ratio (%), median	48.2, (*n* = 23)	17	3.9	0.15
Treatment initiated	24.1 [7/29]	13	6.4	0.60

*Note:* Quantitative anti‐NXP2 titers stratified by major clinical features, including malignancy and organ involvement, are presented. Values are expressed as % [n/N], where N indicates the number of patients assessed for each item. Anti‐NXP2 titers are expressed as medians. Nominal *p* values were calculated using the Mann–Whitney U test.

Nominally significant differences: *p ≤ 0.05.

Patients with dysphagia tended to have higher titers, although the difference did not reach statistical significance (*p* = 0.077). No other nominally significant associations were found between anti‐NXP2 titers and concomitant autoantibodies, any laboratory findings, the presence of individual skin manifestations, electrocardiographic or echocardiographic abnormalities, or treatment interventions.

### 3.6 Longitudinal Changes in Anti‐NXP2 ELISA Titers in Anti‐NXP2‐Positive IIM

Of the anti‐NXP2‐positive myositis patients, five cases had sera stored at multiple time points during follow‐up, allowing the longitudinal assessment of titer dynamics. Treatments included systemic immunosuppressive therapies, such as corticosteroids, in combination with topical therapies. The details were unavailable for one case.

The timing of treatment initiation is indicated by red arrows in Figure 3. Anti‐NXP2 ELISA titers remained within the positive range throughout the observation period, which extended to more than five years, and no seroconversion to negativity was observed. Although titers showed a gradual decreasing trend in some patients, particularly in those with longer follow‐up durations, relatively stable titers were observed in patients with shorter observation intervals. In Pt. 3, the titer re‐increased from 2.97 to 6.48 toward the end of follow‐up. At that time, although the skin eruptions had previously improved, pruritus newly developed and a mild rash was observed on both elbows and the anterior neck.

**FIGURE 3:**
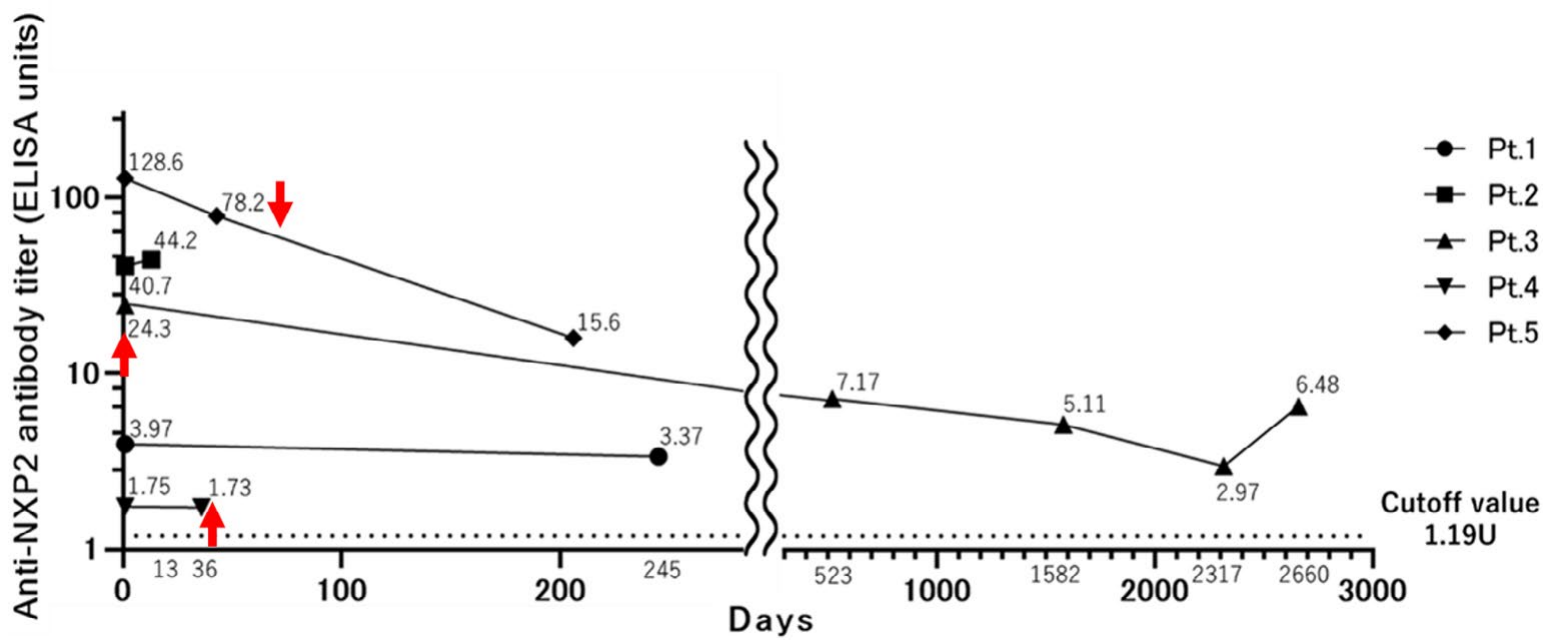
Longitudinal changes in anti‐NXP2 titers in anti‐NXP2‐positive IIM patients Serial anti‐NXP2 measurements by ELISA were performed for individual patients over time. Antibody levels are expressed in ELISA units and plotted on a log10 scale on the y‐axis, whereas the x‐axis represents days elapsed on a linear scale from the initial measurement. Each symbol represents an individual patient (Pt 1–5), and lines connect sequential measurements from the same patient. The dotted horizontal line indicates the pre‐defined cutoff value for anti‐NXP2 positivity. Red arrows indicate the timing of initiation of systemic therapy for IIM or chemotherapy for malignancy. An axis break is used to accommodate long‐term follow‐up.

## 4 Discussion

In this study, we compared anti‐NXP2‐positive IIM and anti‐TIF1‐γ‐positive IIM in a multicenter cohort and established a newly optimized ELISA system that is capable of quantitatively assessing anti‐NXP2 titers. Using this approach, we clarified the shared and distinct features between these two MSA subsets and systematically presented the potential clinical implications of quantitatively measured anti‐NXP2 titers.

The anti‐NXP2‐positive IIM patient group showed a younger age at onset than the anti‐TIF1‐γ‐positive IIM patient group, whereas sex and the frequencies of most cutaneous manifestations were broadly similar. In contrast, classic DM skin manifestations, such as Gottron's sign/papules and heliotrope rash, were significantly more frequent in the anti‐TIF1‐γ‐positive group, supporting the notion that anti‐TIF1‐γ‐positive DM represents a more skin‐dominant clinical phenotype than anti‐NXP2‐positive DM does [8, 22].

Conversely, anti‐NXP2‐positive IIM appears to be characterized by a phenotype in which muscle involvement and systemic disease burden are more prominent. In our cohort, CK and aldolase levels at the initial visit were significantly higher in anti‐NXP2‐positive IIM cases than in anti‐TIF1‐γ‐positive IIM cases. This finding is consistent with previous reports that anti‐NXP2‐positive DM is more likely to be accompanied by severe muscle weakness, and it suggests that substantial muscle damage occurs in this subset [23]. In addition, LDH was also significantly higher in anti‐NXP2‐positive IIM cases than in anti‐TIF1‐γ‐positive IIM cases. While CK and aldolase are considered direct markers of muscle injury in IIM, LDH is a non‐tissue‐specific enzyme and is typically regarded as an ancillary or indirect marker in myositis; LDH has not been clearly emphasized as a defining feature of specific MSA subsets [24]. The concurrent elevation of LDH along with CK and aldolase in anti‐NXP2‐positive IIM may indicate that this subset is associated with broader or more intense tissue involvement reflecting secondary systemic consequences of muscle injury.

With respect to pulmonary and cardiac involvement, there were no differences in ILD prevalence, ILD biomarkers (KL‐6 and SP‐D), or abnormalities on electrocardiography and echocardiography between the anti‐NXP2‐positive and anti‐TIF1‐γ‐positive IIM groups. Prior studies have suggested that anti‐NXP2‐positive and anti‐TIF1‐γ‐positive subsets belong to a relatively low‐risk group for ILD; our findings are consistent with this notion [25]. Notably, the prevalence of ILD was lower in our cohort (13.8% in anti‐NXP2‐positive and 9.7% in anti‐TIF1‐γ‐positive cases) than in some previous reports, and we did not identify a clear trend regarding lung or cardiac involvement between the anti‐NXP2‐positive and anti‐TIF1‐γ‐positive groups.

Regarding malignancy, as in prior studies, malignancy was highly prevalent in anti‐TIF1‐γ‐positive cases (approximately 41%–68%) [26, 27], and 55.2% of the anti‐TIF1‐γ‐positive patients in our cohort met criteria for CAM. In contrast, malignancy was observed in 13.8% of the present anti‐NXP2‐positive cases. Nevertheless, because malignancy occurred at a meaningful frequency in the anti‐NXP2‐positive subset, anti‐NXP2 may be positioned as a major cancer‐associated MSA, after anti‐TIF1‐γ, and it should not be overlooked when cancer risk is assessed in DM.

Based on these group differences, we developed a novel clinical scoring system to distinguish anti‐NXP2‐positive cases from anti‐TIF1‐γ‐positive cases. Importantly, the absence of false positives suggests the potential utility of the scoring system as a tool to support clinical suspicion of anti‐NXP2‐positive disease in clinical practice. The ROC curve analyses used to determine the cutoff values for age at onset, LDH, and CK are shown in Figure S3.

However, because five false‐negative cases were observed, the score cannot capture all anti‐NXP2‐positive cases; antibody testing remains essential for definitive classification. Nonetheless, in settings where testing for non‐insurance‐covered MSAs is difficult, the score may offer clinical value as a simple bedside indicator to raise suspicion for the anti‐NXP2 subset.

An important limitation of the present scoring system is that malignancy status is included as one of its components. Because malignancy status is a time‐dependent variable, the use of this score at the earliest stage of diagnosis may be limited, particularly before baseline malignancy assessment has been completed. Therefore, in clinical practice, this score should be interpreted as an adjunctive tool after the initial evaluation, including malignancy assessment, has been performed. If malignancy is subsequently detected during follow‐up, the malignancy‐related component and the total score may change. In previous reports that included the timing of malignancy diagnosis, malignancies in anti‐NXP2 antibody‐positive and anti‐TIF1‐γ antibody‐positive IIM/DM were often identified before, at the time of, or relatively soon after IIM/DM diagnosis [28, 29]. In the present cohort, malignancies in the anti‐TIF1‐γ antibody‐positive group showed a similar temporal pattern and were diagnosed close to the time of myositis diagnosis, with a median interval of 0.12 months among patients with available timing information. The early temporal clustering observed in the anti‐TIF1‐γ antibody‐positive group supports the clinical validity of the proposed scoring system, because malignancy is a negative scoring component and its early recognition would tend to lower the baseline score, thereby shifting anti‐TIF1‐γ antibody‐positive patients toward a more typical anti‐TIF1‐γ antibody‐positive profile. Nevertheless, subsequent malignancy detection remains possible, particularly in anti‐NXP2 antibody‐positive patients, and currently available data are limited regarding how frequently the malignancy‐related component changes after the initial evaluation. Further studies are needed to clarify how such longitudinal changes affect the performance of the scoring system over time.

In analyses of antibody titers, quantitatively measured anti‐NXP2 titers showed nominally significant positive correlations with ESR_max_ and cardiothoracic ratio, whereas no nominally significant correlations were observed with other features, including CK and aldolase.

Tables 2 and 3 are exploratory and are not designed to test predefined confirmatory hypotheses. Therefore, nominal *p* values are presented without formal correction for multiplicity. Because multiple comparisons were performed, however, the possibility of false‐positive findings cannot be excluded. In sensitivity analyses applying Holm and Benjamini–Hochberg adjustments, the nominally significant findings do not remain statistically significant. Accordingly, these findings should be interpreted cautiously as exploratory associations.

ESR is a nonspecific parameter influenced by multiple factors such as inflammation, anemia, infection, and malignancy [30], and it has been reported not to directly reflect myositis activity in DM [31]. The observed positive association between anti‐NXP2 titers and ESR_max_ may therefore suggest that anti‐NXP2 titers reflect aspects of systemic inflammatory or immunological status, potentially including malignancy‐related inflammation, rather than localized muscle disease activity. Furthermore, the correlation with cardiothoracic ratio may indirectly reflect chronic inflammatory burden affecting the cardiopulmonary system.

Importantly, anti‐NXP2 titers were nominally higher in patients with malignancy in the present cohort. While the association is not as strong as that observed for anti‐TIF1‐γ titers, these results suggest that quantitative anti‐NXP2 titers may provide supporting information when tumor risk is assessed. However, this finding was based on only four patients with malignancy, and it did not remain statistically significant after adjustment for multiple comparisons. Therefore, the association between anti‐NXP2 titers and malignancy should be regarded as a preliminary observation requiring further validation in larger cohorts, rather than as definitive evidence that anti‐NXP2 titers can independently predict malignancy risk. Prior reports on DM/PM have shown that ESR is significantly higher in patients with malignancy, that elevated ESR (> 35 mm/h) predicts malignancy, and that ESR elevation is an independent risk factor in multivariable analyses [32, 33, 34]. Taken together, these findings suggest that careful clinical evaluation for malignancy may be particularly warranted in patients with high anti‐NXP2 titers accompanied by elevated ESR.

In the longitudinal analyses, anti‐NXP2 titers did not become negative even after more than five years of observation. Although titers showed a gradual decline in some patients, particularly in those with longer follow‐up durations, relatively stable titers were observed in patients with shorter observation intervals. In addition, in Pt. 3, the titer re‐increased toward the end of follow‐up, and this increase was accompanied by newly developed pruritus and mild rash, suggesting possible mild cutaneous worsening. However, because follow‐up was discontinued thereafter, whether this represented a definite clinical relapse could not be confirmed. To clarify the relationship between treatment and antibody kinetics, the timing of treatment initiation is indicated by red arrows in Figure 3. These findings suggest that anti‐NXP2 antibodies may persist over time and do not readily become negative after treatment. The observed gradual decline in selected cases is consistent with three cases previously reported by Yuasa et al., in which anti‐NXP2 titers decreased after the initiation of systemic therapy [35].

These findings raise the possibility that anti‐NXP2 titers may change in association with disease course; however, the small number of longitudinally assessed patients, the heterogeneous follow‐up durations, and the limited information regarding standardized timing of treatment initiation and antibody measurement preclude any definitive conclusion regarding their utility as a marker of treatment response. Therefore, the current findings should be interpreted as preliminary and hypothesis‐generating. Further prospective studies with serial antibody measurements at predefined time points, and standardized assessments of disease activity and treatment status are required to determine whether quantitative anti‐NXP2 titers can be used for longitudinal disease assessment.

This study has several limitations. First, anti‐NXP2‐positive IIM is a relatively rare subset, and despite the multicenter retrospective design, the limited sample size may have reduced statistical power. Second, anti‐NXP2 titers were measured using our in‐house ELISA, and the lack of international standardization may limit inter‐laboratory comparability and require cautious interpretation, particularly for borderline cases. Third, clinical data were retrospectively collected, resulting in missing variables and potential selection bias, as some cases were excluded from the scoring analysis. Finally, longitudinal analyses were performed in only five patients with heterogeneous follow‐up durations. Although the timing of treatment initiation is now indicated by red arrows in Figure 3, treatment regimens and the intervals between treatment initiation and antibody measurements were not standardized. While patients with shorter observation intervals demonstrated relatively stable titers, the apparent decrease was mainly observed in cases with extended follow‐up. Therefore, the current findings do not allow definitive conclusions regarding long‐term antibody kinetics nor whether anti‐NXP2 titers reliably reflect treatment response and should be regarded as preliminary and hypothesis‐generating. Further prospective studies are needed to validate the clinical significance of serial quantitative anti‐NXP2 measurements.

In conclusion, in this multicenter cohort study, anti‐NXP2‐positive IIM was characterized by a more muscle‐dominant phenotype compared with anti‐TIF1‐γ‐positive DM. Our newly developed clinical score showed high discriminatory performance and may serve as a useful adjunct when antibody testing is unavailable. The quantitative assessment of anti‐NXP2 titers obtained by our newly optimized ELISA system suggested possible associations with systemic disease burden and malignancy risk. Longitudinal analysis showed that anti‐NXP2 titers remained positive over time and gradually declined in some patients, although the small number of longitudinally assessed cases precludes definitive conclusions regarding their utility as markers of treatment response.

These findings suggest that quantitative anti‐NXP2 evaluation may support clinical stratification and provide complementary information during longitudinal follow‐up, warranting further prospective validation.

## Funding

This work was financially supported by KAKENHI Grants‐in‐Aid for Scientific Research from the Japan Society for the Promotion of Science (JP25K11603) and JST SPRING, Number JPMJSP2125.

## Ethics Statement

This retrospective study was approved by the institutional review board of Nagoya University Graduate School of Medicine (approval no. 2014‐0324‐14). All participants provided written informed consent for study participation, including serum use. The study was conducted in accordance with the Declaration of Helsinki. Registry and Registration No. of the study/trial: N/A. Animal Studies: N/A.

## Conflicts of Interest

Masashi Akiyama is an Editorial Board member of *The Journal of Dermatology* and a co‐author of this article. Kimiko Nakajima is also an Editorial Board member of *The Journal of Dermatology* and a co‐author of this article. To minimize bias, they were excluded from all editorial decision‐making related to the acceptance of this article for publication. The other authors declare no conflicts of interest.

## Supporting information


**Figure S1:** IPP of biotinylated recombinant NXP2 with patients' sera. The input lane contains half the dose (8 μL) of biotinylated recombinant NXP2 protein that was used for the IPP assay. The band in this lane that migrated at approximately 140 kDa corresponds to NXP2. Lane 1 contains the NXP2 immunoprecipitated by the sera from a representative patient who was anti‐NXP2‐positive in ELISA. Lanes 2, 4, 6, and 7 contain the sera that were just above the cutoff level in ELISA. Lanes 8 and 9 are the equivocal sera in ELISA. Lanes 3 and 5 contain the NXP2 immunoprecipitated by the sera of patients that were anti‐NXP2‐negative in ELISA. Lanes 10, 11, and 12 contain sera from three healthy controls. The asterisk denotes biotinylated NXP2 band.
IPP: immunoprecipitation assay.
**Figure S2:** Correlations between anti‐NXP2 titers, ESR, and cardiothoracic ratio. Spearman's rank correlation was used to assess associations between anti‐NXP2 titers and clinical or laboratory parameters. Among laboratory variables, maximum erythrocyte sedimentation rate (ESR_max_) showed a nominally moderate positive correlation with antibody titer (*r* = 0.51, *p* = 0.0090). Cardiothoracic ratio was also nominally positively correlated with antibody titer (*r* = 0.49, *p* = 0.017).
ESR, erythrocyte sedimentation rate; NXP2, nuclear matrix protein 2.
**Figure S3:** ROC curve analyses for determining cutoff values used in the clinical scoring system. ROC curve analyses were performed to determine cutoff values for continuous variables included in the clinical scoring system: (A) age at onset, (B) LDH, and (C) CK. Anti‐NXP2 antibody positivity was used as the outcome. The optimal cutoff value for each variable was selected based on the Youden index, defined as sensitivity + specificity −1. The analyses identified cutoff values of 49 years for age at onset, 423 U/L for LDH, and 367 U/L for CK. Because age was recorded in whole years, the age cutoff was expressed clinically as age at onset < 50 years in the scoring system. The points shown on each ROC curve indicate the selected cutoff values, with specificity and sensitivity shown in parentheses.
CK, creatine kinase; LDH, lactate dehydrogenase; NXP2, nuclear matrix protein 2; ROC, receiver operating characteristic.
**Table S1:** Malignancy profiles and timing of detection in patients with anti‐NXP2 or anti‐TIF1γ antibody‐positive myositis.

## Data Availability

The data that support the findings of this study are available from the corresponding author upon reasonable request. The data are not publicly available due to privacy or ethical restrictions.
