# Supplementary material for: Multicenter Clinical Comparison Between Anti‐NXP2 and Anti‐TIF1‐γ Antibody‐Positive Myositis, and Analysis of the Clinical Significance of Anti‐NXP2 Antibody Titers in Idiopathic Inflammatory Myopathies
Source: J Dermatol. 2026 Sep 16;53(11):1626–37. doi: 10.1111/1346-8138.70490 (PMC13737459; doi:10.1111/1346-8138.70490)
Supplement: Supplementary file 1 [file JDE-53-1626-s001.docx]

**Supplementary information**

・Supplementary data, Supplementary methods

・**Supplementary Figure S1. IPP of biotinylated recombinant NXP2 with patients’ sera**

**・Supplementary Figure S2. Correlations between anti-NXP2 titer, ESR, and** cardiothoracic ratio

**・Supplementary Figure S3. ROC curve analyses for determining cutoff values used in the clinical scoring system**

・**Supplementary Table 1. Malignancy profiles and timing of detection in patients with anti-NXP2 or anti-TIF1γ antibody-positive myositis**

**Supplementary data**

**Supplementary methods**

**Immunoprecipitation (IPP) Assay**

Immunoprecipitation was performed using transcription and translation (TnT) products with minor modifications, as previously described [1–4]. Briefly, 16 μL of patient serum was incubated with 20 μL of 50% slurry Protein G Sepharose 4 Fast Flow (GE Healthcare, Buckinghamshire, UK) and 300 μL of IPP buffer (PBS containing 1% Nonidet P-40) at 4°C for 1 hour. After five washes with IPP buffer, the beads were resuspended in Laemmli sample buffer, and IgG-bound proteins were separated by 7.5% SDS-PAGE. Proteins were transferred onto Immobilon-P membranes (Millipore, Billerica, MA, USA), and biotinylated proteins were detected using Western Blue Substrate (Promega).

**Supplementary Figure S1. IPP of biotinylated recombinant NXP2 with patients’ sera**

The input lane contains half the dose (8 μL) of biotinylated recombinant NXP2 protein that was used for the IPP assay. The band in this lane that migrated at approximately 140 kDa corresponds to NXP2. Lane 1 contains the NXP2 immunoprecipitated by the sera from a representative patient who was anti-NXP2-positive in ELISA. Lanes 2, 4, 6, and 7 contain the sera that were just above the cutoff level in ELISA. Lanes 8 and 9 are the equivocal sera in ELISA. Lanes 3 and 5 contain the NXP2 immunoprecipitated by the sera of patients that were anti-NXP2-negative in ELISA. Lanes 10, 11, and 12 contain sera from three healthy controls. The asterisk denotes biotinylated NXP2 band.

IPP: immunoprecipitation assay.

**Supplementary Figure S2. Correlations between anti-NXP2 titers, ESR, and cardiothoracic ratio**

Spearman’s rank correlation was used to assess associations between anti-NXP2 titers and clinical or laboratory parameters. Among laboratory variables, maximum erythrocyte sedimentation rate (ESR_max_) showed a nominally moderate positive correlation with antibody titer (r = 0.51, P = 0.0090). Cardiothoracic ratio was also nominally positively correlated with antibody titer (r = 0.49, P = 0.017).

ESR, erythrocyte sedimentation rate; NXP2, nuclear matrix protein 2.

**Supplementary Figure S3. ROC curve analyses for determining cutoff values used in the clinical scoring system**

ROC curve analyses were performed to determine cutoff values for continuous variables included in the clinical scoring system: (A) age at onset, (B) LDH, and (C) CK. Anti-NXP2 antibody positivity was used as the outcome. The optimal cutoff value for each variable was selected based on the Youden index, defined as sensitivity + specificity − 1. The analyses identified cutoff values of 49 years for age at onset, 423 U/L for LDH, and 367 U/L for CK. Because age was recorded in whole years, the age cutoff was expressed clinically as age at onset <50 years in the scoring system. The points shown on each ROC curve indicate the selected cutoff values, with specificity and sensitivity shown in parentheses.

CK, creatine kinase; LDH, lactate dehydrogenase; NXP2, nuclear matrix protein 2; ROC, receiver operating characteristic.

**Supplementary Table 1. Malignancy profiles and timing of detection in patients with anti-NXP2 or anti-TIF1γ antibody-positive myositis.**

|  | Cancer | | Months^†^ |  |
| --- | --- | --- | --- | --- |
| anti-NXP2^+^  Pt. 1  Pt. 2  Pt. 3  Pt. 4  anti-TIF1~~-~~γ^+^  Pt. 1  Pt. 2  Pt. 3  Pt. 4  Pt. 5  Pt. 6  Pt. 7  Pt. 8  Pt. 9  Pt. 10  Pt. 11  Pt. 12  Pt. 13  Pt. 14  Pt. 15  Pt. 16  Pt. 17  Pt. 18  Pt. 19  Pt. 20  Pt. 21 | Ovarian  Endometrial  Bladder CIS  Lung  Breast  Bladder  Ovarian  Gallbladder  Small-cell Lung  Gastric  Prostate  Breast  Gastric  Gastric  Small-cell lung  Gastric  Gastric  Esophageal  Breast  Rectal  Gastric  Gastric  Breast  Gastric  Gastric | | +0.39  +17.97  +19.02  +0.92  −0.82 -31.34 +0.85 +45.40 +0.30 +0.20 −23.26 +0.76 −2.30 +0.92 +0.03 +12.55 N.A. N.A. −15.15 −1.00 −0.43 +0.46 N.A. N.A. N.A. |  |
| Autoantibody type | | Interval from IIM/DM diagnosis to  cancer diagnosis, months (IQR) | | |
| anti-NXP2^+^  anti-TIF1~~-~~γ^+^ | | 9.45 (0.79–18.23)  0.12 (−1.33–0.78) | | |

^†^Months from myositis diagnosis to cancer diagnosis. Positive values indicate cancer diagnosis after myositis diagnosis, and negative values indicate cancer diagnosis before myositis diagnosis. Patients for whom the exact timing was unavailable were excluded from the calculation. CIS, carcinoma in situ. N.A., not available.

In the anti-NXP2 antibody-positive cohort, malignancy was identified in four patients during follow-up after myositis diagnosis. The median interval from myositis diagnosis to malignancy diagnosis was 9.45 months (IQR, 0.79–18.23 months). The cancer types were ovarian cancer, endometrial cancer, bladder carcinoma in situ, and lung cancer.

In the anti-TIF1-γ antibody-positive cohort, malignancy was more frequently observed and tended to cluster around the time of myositis diagnosis. Among patients with available information on the timing of malignancy diagnosis, the median interval from myositis diagnosis to malignancy diagnosis was 0.12 months (IQR, −1.33–0.78 months), where negative values indicate cancer diagnosis before myositis diagnosis. The exact timing of malignancy diagnosis was unavailable for the remaining patients. The cancer types consisted of breast cancer, bladder cancer, ovarian cancer, gallbladder cancer, small-cell lung cancer, gastric cancer, prostate cancer, esophageal cancer, and rectal cancer. These findings are summarized in Supplementary Table 1.

**References**

1	Y. Ogawa, K. Sugiura, A. Watanabe, et al., “Autoantigenicity of DFS70 is restricted to the conformational epitope of C-terminal alpha-helical domain,” *J Autoimmun* 23 (2004): 221–231, https://doi.org/10.1016/j.jaut.2004.07.003.

2	M. Okamoto, Y. Ogawa, A. Watanabe, et al., “Autoantibodies to DFS70/LEDGF are increased in alopecia areata patients,” *J Autoimmun* 23 (2004): 257–266, https://doi.org/10.1016/j.jaut.2004.07.004.

3	N. Goto, K. Sugiura, Y. Ogawa, et al., “Anti-p80 coilin autoantibodies react with a conserved epitope and are associated with anti-DFS70/LEDGF autoantibodies,” *J Autoimmun* 26 (2006): 42–51, https://doi.org/10.1016/j.jaut.2005.09.001.

4	N. Tanaka, Y. Muro, K. Sugiura, and Y. Tomita, “Anti-SS-A/Ro antibody determination by indirect immunofluorescence and comparison of different methods of anti-nuclear antibody screening: evaluation of the utility of HEp-2 cells transfected with the 60 kDa SS-A/Ro as a substrate,” *Mod Rheumatol* 18 (2008): 585–592, https://doi.org/10.1007/s10165-008-0100-x.
